# Surgical Outcome of Iris Claw and Scleral Tuck Intraocular Lens in Primary Cataract Surgery by Phacoemulsification in a Tertiary Care Center: Protocol for a Randomized Controlled Trial

**DOI:** 10.2196/80943

**Published:** 2026-02-23

**Authors:** Mohammed Kaderi, Sachin Daigavane

**Affiliations:** 1 Department of Ophthalmology, Acharya Vinoba Bhave Rural Hospital, Jawaharlal Nehru Medical College, Datta Meghe Institute of Higher Education and Research, Sawangi, Wardha, Maharashtra, 442001, India, 91 8698786925

**Keywords:** retropupillary iris claw, scleral tuck lens, cataract surgery, intraocular lens, phacoemulsification, surgical outcome, blindness

## Abstract

**Background:**

Cataract remains the leading cause of reversible blindness worldwide, particularly in older adults, in whom capsular weakness during phacoemulsification may preclude posterior chamber intraocular lens (IOL) implantation. In such scenarios, alternative fixation methods such as scleral-sutured and iris-claw lenses are preferred. However, direct comparative evidence regarding their safety, stability, and postoperative visual outcomes during primary cataract surgery is limited.

**Objective:**

This study aims to compare the surgical and visual outcomes between retropupillary iris-claw IOL implantation and scleral-sutured fixation of single-piece foldable IOLs performed during primary cataract surgery by phacoemulsification in patients with inadequate capsular support.

**Methods:**

This is a prospective, 2-arm, open-label randomized controlled trial being conducted at the Department of Ophthalmology, Acharya Vinoba Bhave Rural Hospital, Sawangi, India. A total of 92 patients aged 35 to 75 years with intraoperative posterior capsular compromise will be randomized (block randomization, sealed opaque envelopes) into 2 groups: group A (retropupillary iris-claw IOL) and group B (scleral-sutured IOL). Preoperative, intraoperative, and postoperative data will be collected at day 1, day 15, day 30, and day 90. The primary outcomes are best-corrected visual acuity and lens-induced astigmatism at 3 months. Secondary outcomes include IOL centration or tilt, intraoperative and postoperative complications, and patient satisfaction (assessed using the National Eye Institute Visual Function Questionnaire-25). Data will be analyzed using ANOVA and mixed-effects models.

**Results:**

Recruitment commenced in March 2025 and is expected to conclude by September 2025, with interim data analysis scheduled for November 2025. As of the current submission, patient enrollment is ongoing, and no interim results have been analyzed.

**Conclusions:**

This randomized protocol is designed to generate comparative clinical evidence on the efficacy, safety, and visual stability of iris-claw and scleral-sutured IOL fixation in primary cataract surgery. The findings will contribute to optimizing surgical decision-making in cases with deficient posterior capsular support.

## Introduction

### Overview

Cataract remains the leading cause of reversible blindness worldwide and continues to impose a major public health burden, particularly among older adults in low- and middle-income countries [1]. In 2020, an estimated 79 million individuals aged 50 years and older were affected globally, representing a 39% increase in prevalence since 1993 [2]. The only definitive treatment is surgical extraction of the opacified lens followed by intraocular lens (IOL) implantation [3]. Phacoemulsification with posterior chamber IOL placement is the preferred modern technique due to its faster recovery, smaller incision size, and superior visual outcomes [4]. However, intraoperative or pre-existing capsular rupture can complicate surgery and preclude standard posterior chamber fixation, resulting in aphakia and unstable visual rehabilitation [3,5].

When the posterior capsule is compromised, secondary fixation techniques are required to achieve optical stability. The 2 widely practiced approaches are retropupillary iris-claw IOL fixation and scleral-sutured posterior chamber IOL fixation [6]. In iris-claw implantation, the lens is enclavated onto the midperipheral iris, providing a relatively simple and reproducible procedure with shorter operative time [5]. Conversely, scleral-sutured fixation secures the IOL haptics within scleral flaps using 10‐0 polypropylene (Prolene; Ethicon) sutures, offering stable posterior chamber positioning and a reduced risk of endothelial trauma but requiring greater surgical expertise [7]. Both techniques have evolved substantially, yet each carries distinct risks, such as iris chafing, pupil distortion, or suture degradation [8,9].

Comparative evidence between these 2 fixation methods primarily originates from retrospective or heterogeneous secondary IOL implantation studies [7,8]. A limited number of prospective randomized studies have evaluated their performance in primary cataract surgeries where capsular support is inadequate at the index operation. Existing studies report conflicting outcomes: some suggest lower intraoperative complications with iris-claw IOLs [7], while others demonstrate superior centration and long-term stability with scleral fixation [8,9]. Moreover, data on lens-induced astigmatism, patient-reported visual satisfaction, and postoperative quality of life remain scarce. The choice between scleral-sutured and retropupillary iris-claw fixation in primary cataract surgery requires solid evidence on their safety, effectiveness, and patient experience. This randomized trial addresses that gap by directly comparing both techniques under standardized surgical conditions. It will assess visual outcomes, complications, and patient-reported satisfaction to help guide reliable IOL selection when posterior capsular support is inadequate.

### Research Question

Among adult patients undergoing primary cataract surgery by phacoemulsification with inadequate posterior capsular support, does retropupillary iris-claw IOL implantation provide comparable or superior visual and surgical outcomes compared with scleral-sutured fixation of single-piece foldable IOLs?

### Hypothesis

This randomized controlled trial hypothesizes that retropupillary iris-claw IOL implantation will achieve visual outcomes (measured by best-corrected visual acuity [BCVA] and lens-induced astigmatism at 3 months) that are noninferior or superior to those obtained with scleral-sutured fixation. It is further hypothesized that the iris-claw technique will demonstrate shorter operative time and fewer intraoperative and postoperative complications, while maintaining comparable centration and patient-reported satisfaction scores.

## Methods

### Study Design

This will be a prospective, hospital-based, 2-arm, open-label randomized controlled trial designed to compare the surgical and visual outcomes between retropupillary iris-claw IOL implantation and scleral-sutured fixation of single-piece foldable IOLs in patients undergoing primary cataract surgery by phacoemulsification. The trial will be conducted at the Department of Ophthalmology, Acharya Vinoba Bhave Rural Hospital (AVBRH), Sawangi, Wardha, India.

### Study Setting

All surgical and postoperative procedures will be performed in the dedicated ophthalmic operation theaters of AVBRH under standardized aseptic conditions by trained consultant ophthalmic surgeons. Each participating surgeon will have independently performed at least 30 cases of both iris-claw and scleral-sutured IOL fixation techniques to ensure procedural consistency.

### Study Population

Patients aged between 35 and 75 years scheduled for primary cataract surgery by phacoemulsification, in whom intraoperative posterior capsular rupture or deficient zonular support precludes in-the-bag IOL implantation, will be considered for inclusion. Inadequate posterior capsular support was defined as one or more of the following intraoperative conditions: posterior capsular rupture with vitreous disturbance, zonular dialysis exceeding 3 clock hours, or combined zonular-capsular instability preventing safe in-the-bag fixation. All surgeons will follow this standardized definition to ensure uniform eligibility.

### Eligibility Criteria

Inclusion and exclusion criteria for study participation are summarized in Textbox 1 (see Multimedia Appendix 1 for the sample size calculation).

Textbox 1. **Inclusion and exclusion criteria of the study population.**

**Inclusion Criteria**

- Patients aged 35 to 75 years undergoing primary cataract surgery by phacoemulsification
- Presence of inadequate posterior capsular support identified intraoperatively, including posterior capsular rupture with vitreous disturbance or zonular dialysis exceeding 3 clock hours
- Inability to safely implant an in-the-bag IOL due to capsular or zonular instability
- Both male and female patients
- Willingness to provide written informed consent
- Ability and willingness to comply with postoperative follow-up schedule

**Exclusion Criteria**

- Pre-existing corneal diseases such as keratitis, corneal dystrophy, or corneal opacities
- History of chronic or recurrent uveitis
- Advanced glaucoma (intraocular pressure greater than 21 mm Hg despite medication or optic nerve cup-to-disc ratio greater than 0.7)
- Retinal pathologies, including diabetic retinopathy, hypertensive retinopathy, age-related macular degeneration, or retinitis pigmentosa
- Traumatic aphakia or lens subluxation secondary to ocular trauma
- Active ocular infection or uncontrolled systemic disease likely to impair wound healing

### Randomization, Allocation Concealment, and Blinding

Eligible patients will be randomly allocated in a 1:1 ratio into 2 groups: group A (retropupillary iris-claw IOL) and group B (scleral-sutured fixation). A computer-generated block randomization sequence (block size of 4) will be prepared by an independent statistician. The group assignment will be concealed in sequentially numbered, opaque, sealed envelopes opened only after capsular deficiency confirmation during surgery.

Due to the nature of the interventions, blinding of surgeons and patients will not be feasible. However, postoperative outcome assessors and data analysts will remain blinded to the intervention group to minimize observer bias. Postoperative assessors will follow standardized grading protocols and will remain masked to group assignment to reduce detection bias.

### Intervention Procedures

Both procedures will be performed under peribulbar anesthesia using a standardized phacoemulsification technique.

For group A, a retropupillary iris-claw IOL will be implanted using standard enclavation of haptics onto the posterior iris surface after thorough anterior vitrectomy. For group B, a single-piece foldable IOL will be fixated with 10‐0 polypropylene (Prolene) sutures passed through partial-thickness scleral flaps created at the 3- and 9-o’clock positions, ensuring equal centration. All surgeries will adhere to uniform intraoperative and postoperative protocols, including the use of balanced salt solution, intracameral antibiotics, and a topical steroid–antibiotic combination therapy postsurgery. To ensure reproducibility, procedural videos will be periodically reviewed by the data monitoring committee for intersurgeon standardization.

### Timeline of Assessments

The schedule of preoperative, intraoperative, and postoperative assessments is outlined in Table 1.

**Table 1.** Timeline of clinical assessments and outcome measures.

Study phase	Assessment parameters
Preoperative	Visual acuity (Snellen’s chart), slit-lamp biomicroscopy, intraocular pressure (NCT^a^), fundus evaluation, and biometry (A-scan, keratometry)
Day 0 (surgery)	Type of IOL^b^ fixation, intraoperative complications, surgical duration, and intraoperative stability
Day 1	Corneal clarity, anterior chamber reaction, intraocular pressure, and early complications
Day 15	BCVA^c^, astigmatism (autorefractometry), anterior chamber reaction, and IOL position
Day 30	BCVA, lens centration/tilt (slit-lamp retroillumination as per Calzetti et al [10]), intraocular pressure, and patient comfort
Day 90	Final BCVA, lens-induced astigmatism, postoperative complications, and patient satisfaction (NEI VFQ-25^d^)

a NCT: noncontact tonometry.

b IOL: intraocular lens.

c BCVA: best-corrected visual acuity.

d NEI VFQ-25: National Eye Institute Visual Function Questionnaire-25.

### Outcome Measures

Primary outcomes:

1. BCVA at 3 months using Snellen’s chart, converted to logMAR values for statistical analysis.
2. Lens-induced astigmatism, measured through keratometry and autorefractometry.

Secondary outcomes:

1. Intraoperative complications (posterior capsular rupture extension, vitreous loss, surgical duration).
2. Postoperative complications (hyphema, pigment dispersion, raised intraocular pressure, IOL decentration, cystoid macular edema).
3. IOL centration and tilt, assessed by slit-lamp retroillumination following the method of Calzetti et al [10].
4. Patient-reported satisfaction and visual function, measured using the National Eye Institute Visual Function Questionnaire-25 at 3 months.

### Adverse Event Reporting and Monitoring

All adverse events (AEs) will be documented at each follow-up. Serious AEs will be reported to the Institutional Ethics Committee within 7 days and nonserious AEs within 30 days. A data monitoring committee comprising 2 ophthalmologists and 1 biostatistician will perform quarterly reviews of safety, recruitment progress, and protocol compliance. Any protocol amendments will require prior institutional ethics committee approval and updating of the Clinical Trial Registry of India.

### Data Management and Statistical Analysis

Statistical methods used for data management and analysis are summarized in this section. Continuous variables (eg, BCVA, astigmatism, and intraocular pressure) will be summarized as mean (SD) and analyzed using the independent *t* test for between-group comparisons and repeated-measures ANOVA for longitudinal analysis. Categorical variables (eg, complications and sex) will be compared using the chi-square test or the Fisher exact test. Multivariate analysis will be performed using mixed-effects linear regression to adjust for baseline covariates (age and preoperative vision). Missing data will be handled using multiple imputation under a missing-at-random assumption. Interim analysis will be conducted after 50% enrollment to evaluate safety and adherence; no stopping rules for efficacy are planned. Statistical analyses will be performed using SPSS software (version 27.0; IBM Corp). A *P* value <.05 will be considered statistically significant.

### Ethical Considerations

The study was approved by the Institutional Ethics Committee of Datta Meghe Institute of Higher Education and Research (DMIHER(DU)/IEC/2024/24, dated May 3, 2024) and was prospectively registered with the Clinical Trial Registry of India (CTRI/2025/03/083687) on March 28, 2025. The trial will be conducted in accordance with the Declaration of Helsinki and Good Clinical Practice guidelines. Written informed consent will be obtained from all participants prior to enrollment. Participant privacy and confidentiality will be maintained, and all data will be deidentified prior to analysis and publication. Data will be recorded in standardized case report forms and entered into a secure password-protected database. All study data will be anonymized prior to analysis and dissemination. Personally identifiable information, including names and medical record numbers, will be removed or replaced with coded identifiers. The reidentification key will be stored separately in a secure, password-protected database accessible only to the principal investigator.

## Results

Patient recruitment began in March 2025 at the Department of Ophthalmology, AVBRH, Sawangi, India, with an estimated completion date of September 2025. As of this submission, participant enrollment and follow-up are ongoing, and no interim data have been analyzed. Preliminary observations indicate adherence to the standardized surgical protocol and follow-up schedule. Data collection for all preoperative, intraoperative, and postoperative variables, including BCVA, IOL centration, and patient-reported satisfaction, will continue as per the CONSORT flow framework. Final analysis and dissemination of results are expected by early 2026.

## Discussion

Management of aphakia during cataract surgery remains a critical surgical challenge, particularly when posterior capsular rupture or zonular instability precludes in-the-bag IOL implantation [11,12]. Although several fixation techniques have been developed, the comparative efficacy and safety of scleral-sutured and retropupillary iris-claw fixation during primary cataract surgery remain insufficiently explored [13]. This protocol aims to prospectively evaluate these two commonly used methods under standardized conditions. The rationale for comparing scleral-sutured and iris-claw fixation stems from their distinct anatomical and procedural advantages. Retropupillary iris-claw lenses offer a posterior chamber position similar to that of an in-the-bag IOL while avoiding contact with the corneal endothelium and anterior chamber angle [2,3]. They are relatively simple to implant, require shorter surgical time, and provide satisfactory centration with minimal postoperative astigmatism [5,7]. Conversely, scleral-sutured fixation restores a near-physiological IOL position behind the iris plane and has been associated with excellent visual outcomes and long-term stability [8]. However, it demands greater surgical skill and carries the potential of suture-related complications, including knot erosion and late dislocation [14,15].

Several retrospective and comparative studies have examined the outcomes of these techniques, but most involve heterogeneous populations or secondary IOL implantations. Madhivanan et al [5] demonstrated that both retropupillary iris-claw and scleral-fixated IOLs achieve significant improvement in BCVA, although scleral fixation required longer surgical duration. Similarly, Durmus et al [7] reported comparable visual outcomes but found that endothelial cell loss was lower with iris-claw lenses than with sutureless scleral fixation. Shahid et al [8] emphasized that scleral fixation provides superior long-term centration yet remains technically demanding and time-intensive. These findings justify the need for a prospective, randomized evaluation focusing specifically on primary cataract cases rather than secondary or complicated aphakia.

This trial’s methodological design addresses previous limitations by incorporating randomization, allocation concealment, and masked outcome assessment. Objective parameters such as IOL tilt and decentration will be evaluated using slit-lamp retroillumination and validated anterior-segment optical coherence tomography methods described by Calzetti et al [10], ensuring quantitative reproducibility. In addition, the inclusion of patient-reported outcomes using the National Eye Institute Visual Function Questionnaire-25 will provide valuable insights into subjective satisfaction and functional vision, which have often been underrepresented in earlier studies.

Ethical oversight, AE monitoring, and quarterly data review by an independent committee have been included to maintain methodological rigor and compliance with Good Clinical Practice guidelines. The statistical plan using mixed-effects modeling and repeated-measures ANOVA will allow robust analysis of longitudinal data while accounting for missing values. The trial does not include an economic analysis, as cost-effectiveness was beyond the scope of the present protocol; however, results regarding operative time and complication rates may have indirect implications for resource optimization in tertiary-care settings. Potential limitations of this study include its single-center design and limited sample size, which may influence external generalizability. Nonetheless, the use of standardized surgical techniques, strict eligibility criteria, and a uniform postoperative evaluation schedule are expected to enhance internal validity and reproducibility.

## Supplementary material

10.2196/80943 Multimedia Appendix 1 Sample size calculation.

10.2196/80943 Checklist 1 SPIRIT checklist.

## Abbreviations

AE: adverse event
AVBRH: Acharya Vinoba Bhave Rural Hospital
BCVA: best-corrected visual acuity
IOL: intraocular lens
