# Supplementary material for: Surgical Outcome of Iris Claw and Scleral Tuck Intraocular Lens in Primary Cataract Surgery by Phacoemulsification in a Tertiary Care Center: Protocol for a Randomized Controlled Trial
Source: JMIR Res Protoc. 2026 Feb 23;15:e80943. doi: 10.2196/80943 (PMC12928542; doi:10.2196/80943)

**Multimedia Appendix 1: Sample Size Calculation**

To calculate the sample size, we used the formula for comparing two independent means. The assumptions included the expected effect size for the difference in BCVA (logMAR) between the two groups, the standard deviation based on previous studies, and the clinically meaningful difference that the study aims to detect. The calculation was performed with a power of 80% and a two-sided alpha level of 0.05. Using these parameters, the minimum required sample size was determined to be 92 participants, with 46 patients allocated to each study arm.


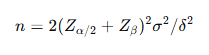


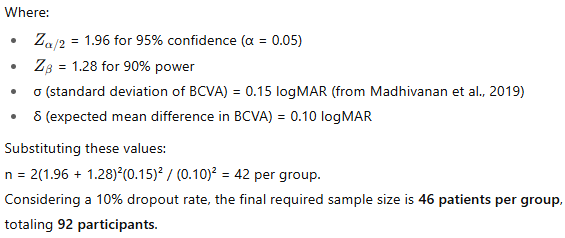

Supplement: Multimedia Appendix 1 [file resprot-v15-e80943-s001.docx]
